# Volumetric and diffusion MRI abnormalities associated with dysarthria in multiple sclerosis

**DOI:** 10.1093/braincomms/fcae177

**Published:** 2024-05-21

**Authors:** Katherine H Kenyon, Myrte Strik, Gustavo Noffs, Angela Morgan, Scott Kolbe, Ian H Harding, Adam P Vogel, Frederique M C Boonstra, Anneke van der Walt

**Affiliations:** Department of Neuroscience, School of Translational Medicine, Monash University, Melbourne, VIC 3004, Australia; Centre for Neuroscience of Speech, University of Melbourne, Parkville, VIC 3052, Australia; Spinoza Centre for Neuroimaging, Netherlands Institute for Neuroscience, Royal Academy for Arts and Sciences, KNAW, Amsterdam 1105 BK, The Netherlands; Melbourne Brain Centre Imaging Unit, Department of Radiology, University of Melbourne, Parkville, VIC 3052, Australia; Department of Neuroscience, School of Translational Medicine, Monash University, Melbourne, VIC 3004, Australia; Centre for Neuroscience of Speech, University of Melbourne, Parkville, VIC 3052, Australia; Department of Neurology, Royal Melbourne Hospital, Parkville, VIC 3052, Australia; Redenlab Inc, Melbourne, VIC 3000, Australia; Murdoch Children’s Research Institute, Genomic Medicine, Speech and Language Group, Parkville 3052, Australia; Department of Speech Pathology and Audiology, University of Melbourne, Parkville 3052, Australia; Department of Neuroscience, School of Translational Medicine, Monash University, Melbourne, VIC 3004, Australia; Department of Neuroscience, School of Translational Medicine, Monash University, Melbourne, VIC 3004, Australia; Centre for Neuroscience of Speech, University of Melbourne, Parkville, VIC 3052, Australia; Melbourne Brain Centre Imaging Unit, Department of Radiology, University of Melbourne, Parkville, VIC 3052, Australia; Redenlab Inc, Melbourne, VIC 3000, Australia; Division of Translational Genomics of Neurodegenerative Diseases, Hertie Institute for Clinical Brain Research, University of Tübingen, Tübingen 72076, Germany; Center for Neurology, University Hospital Tübingen, Tübingen 72076, Germany; The Bionics Institute, East Melbourne, VIC 3002, Australia; Department of Neuroscience, School of Translational Medicine, Monash University, Melbourne, VIC 3004, Australia; Department of Neuroscience, School of Translational Medicine, Monash University, Melbourne, VIC 3004, Australia; Spinoza Centre for Neuroimaging, Netherlands Institute for Neuroscience, Royal Academy for Arts and Sciences, KNAW, Amsterdam 1105 BK, The Netherlands; The Bionics Institute, East Melbourne, VIC 3002, Australia

**Keywords:** multiple sclerosis, diffusion MRI, dysarthria, neurolinguistics

## Abstract

Up to half of all people with multiple sclerosis experience communication difficulties due to dysarthria, a disorder that impacts the motor aspects of speech production. Dysarthria in multiple sclerosis is linked to cerebellar dysfunction, disease severity and lesion load, but the neuroanatomical substrates of these symptoms remain unclear. In this study, 52 participants with multiple sclerosis and 14 age- and sex-matched healthy controls underwent structural and diffusion MRI, clinical assessment of disease severity and cerebellar dysfunction and a battery of motor speech tasks. Assessments of regional brain volume and white matter integrity, and their relationships with clinical and speech measures, were undertaken. White matter tracts of interest included the interhemispheric sensorimotor tract, cerebello-thalamo-cortical tract and arcuate fasciculus, based on their roles in motor and speech behaviours. Volumetric analyses were targeted to Broca’s area, Wernicke’s area, the corpus callosum, thalamus and cerebellum. Our results indicated that multiple sclerosis participants scored worse on all motor speech tasks. Fixel-based diffusion MRI analyses showed significant evidence of white matter tract atrophy in each tract of interest. Correlational analyses further indicated that higher speech naturalness—a perceptual measure of dysarthria—and lower reading rate were associated with axonal damage in the interhemispheric sensorimotor tract and left arcuate fasciculus in people with multiple sclerosis. Axonal damage in all tracts of interest also correlated with clinical scales sensitive to cerebellar dysfunction. Participants with multiple sclerosis had lower volumes of the thalamus and corpus callosum compared with controls, although no brain volumetrics correlated with measures of dysarthria. These findings indicate that axonal damage, particularly when measured using diffusion metrics, underpin dysarthria in multiple sclerosis.

## Teaser

Kenyon *et al*. report on macro- and microstructural injury associated with dysarthria in multiple sclerosis. They show that speech dysfunction in people with multiple sclerosis is more closely linked to microstructural damage within speech-specific white matter tracts than macrostructural atrophy of motor speech areas of the brain.

## Graphical Abstract

**Graphical Abstract:**
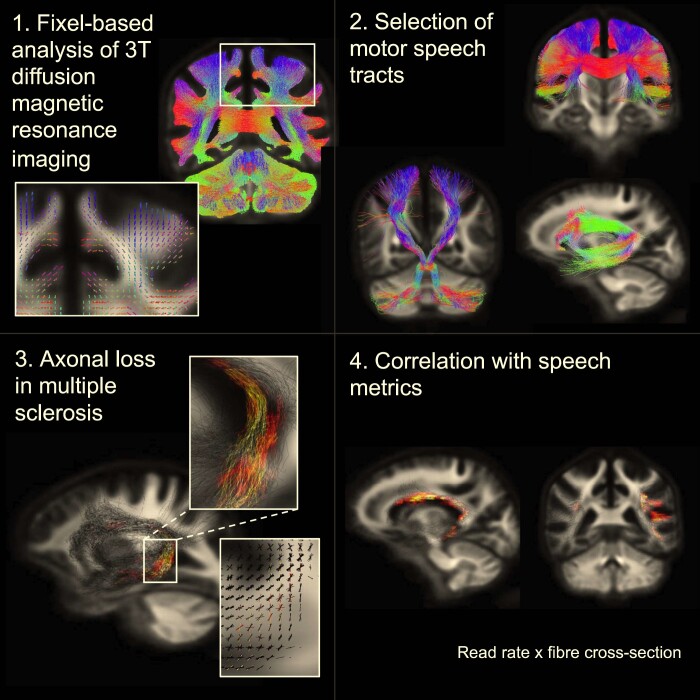

## Introduction

Dysarthria is a motor speech disorder that manifests in 40–50% of persons with multiple sclerosis.^1^ Many persons with multiple sclerosis with dysarthria find that difficulty communicating impacts their ability to engage in social situations,^2^ resulting in a negative self-image, feelings of isolation and decreased quality of life.^3^ Increased severity and frequency of dysarthric symptoms are associated with higher disability in persons with multiple sclerosis,^4^ although they can occur at any disease stage or duration.^5^ Speech analysis can distinguish between healthy people and persons with multiple sclerosis regardless of the level of disability.^6^ Despite the impact of speech difficulties in multiple sclerosis, the core mechanisms of speech dysfunction in persons with multiple sclerosis are not well understood.

The underlying speech pathology in persons with multiple sclerosis has been explored using structural MRI and speech analysis. Acoustic speech analysis allows for more objective and quantitative investigation of speech over perceptual measures. It includes the assessment of speech sound wave properties, such as fundamental frequency and imprecise articulation.^7^ Rusz *et al*.^8^ determined that, in persons with multiple sclerosis with dysarthria, slower articulation rate correlated with reduced grey and white matter fraction and whole-brain volume. Further, this study showed that syllable repetition rate correlated with indicators of cerebellar atrophy.^8^ Noffs *et al*.^4^ demonstrated that white matter volume is associated with speech naturalness and intelligibility. Moreover, an acoustic speech measure of cerebellar dysfunction has been associated with cerebellar white matter volume and cerebellar lesion load.^9^ This research highlights the association between whole-brain grey and white matter loss and measures of dysarthria in persons with multiple sclerosis. However, these studies have focused more on whole-brain or cerebellum volumetrics, and the relative involvement of specific cortical and subcortical brain regions involved in motor speech dysfunction in multiple sclerosis remains unknown. The speech network in the brain includes Broca’s area [left Brodmann areas 44 and 45 (BA44 and BA45)],^10,11^ Wernicke’s area (left BA22),^12,13^ regions within the right cerebellum,^14,15^ orofacial motor cortex, thalamus and basal ganglia.^16,17^ In addition to playing a role in speech production, many of these regions can be affected in multiple sclerosis,^8,18-26^ but have not been assessed with respect to speech in this cohort.

White matter degeneration, driven by demyelination and axonal damage, is a major substrate of functional decline in multiple sclerosis.^27,28^ Past diffusion MRI (dMRI) studies have focused on damage to specific white matter tracts associated with motor performance and language throughout the brain, such as the corpus callosum.^29-32^ Damage to the corpus callosum and connecting sensorimotor tracts—the major interhemispheric sensorimotor pathway (IHST)—is associated with disability progression in multiple sclerosis.^30^ Another specific tract involved in speech is the arcuate fasciculus,^33-35^ which connects BA44/BA45 and BA22, regions involved in speech preparation, production and comprehension.^34-37^ While arcuate fasciculus damage has been recorded in multiple sclerosis, this has been relative to cognitive decline^38,39^ and depression^40^ rather than speech. Finally, damage to the cerebello-thalamo-cortical (CTC) tracts is linked to worse motor performance in persons with multiple sclerosis.^28,41,42^ Both cerebellar and thalamic damage are linked to disordered speech seen in multiple sclerosis.^9,43^ Damage within tracts connecting the left cerebral cortex and right cerebellum has specifically been implicated in poorer performance on language tasks in stroke survivors with aphasia.^44^

Indicators of axonal damage include reduced fibre density (FD) and fibre cross-sectional area within white matter tracts of the brain.^45^ While reduced fibre cross-section is suggestive of tract atrophy, FD estimates the state of remaining white matter tissue.^46^ Fixel-based analysis (FBA) is an advanced dMRI analysis technique that quantifies these measures. Importantly, FBA models the presence of multiple fibre populations, or crossing fibres, within white matter voxels. This allows for more accurate and interpretable findings than standard diffusion imaging measures such as fractional anisotropy.^46^ FBA has recently been used to assess white matter damage in multiple sclerosis. Boonstra *et al*.^28^ observed reductions in FD and FD modulated by fibre cross-section (FD × fibre cross-section, or FDC), indicative of axonal loss, in persons with multiple sclerosis over a 1-year period. Further, lower fibre cross-section in the cingulum, cortico-spinal tract, corpus callosum and cerebellum is seen in progressive multiple sclerosis compared with relapsing–remitting multiple sclerosis, suggesting further damage in more advanced disease.^32,47^ Reduced FDC throughout the cerebellum has been associated with cerebellar dysfunction, lower cerebellar white matter volume and higher cerebellar lesion load in persons with multiple sclerosis.^48^ Additionally, significant reductions in fixel-based measures in the IHST, CTC and corticospinal tract correlate with upper limb motor dysfunction and gait disturbance in persons with multiple sclerosis.^42^ An increasing body of research now supports the link between diffuse white matter damage and motor deficits in persons with multiple sclerosis. However, the specific neural underpinnings of dysarthria in this population remain unclear.

The present study therefore aimed to determine the pathophysiology of speech in persons with multiple sclerosis using diffusion and T_1_-weighted MRI. We aimed to identify dMRI markers of axonal loss using FBA, to assess structural changes using regional volumetric analyses and to determine associations between these brain measures and speech dysfunction in persons with multiple sclerosis. Based on previous research, we expect to find evidence of axonal damage in motor speech tracts of persons with multiple sclerosis, as indicated by lower fixel-based metrics of FD and cross-section compared with healthy controls (HCs). Motor speech regions of the brain will also show evidence of atrophy in persons with multiple sclerosis as measured using volumetric analysis of T_1_-weighted MRI. We further hypothesize that these findings will correlate with measures of dysarthria.

## Materials and methods

### Participants

Fifty-two persons with multiple sclerosis (mean age = 47.8 years, SD age = 11.2 years, 71% female) and 14 age- and sex-matched HCs (mean age = 44.5 years, SD age = 14.5 years, 74% female) were recruited to participate in this study. Multiple sclerosis participants were able to continue their usual disease-modifying therapies for the duration of this study. Exclusion criteria were a multiple sclerosis relapse requiring treatment with corticosteroids in the 3 months prior to study enrolment or exposure to onabotulinum toxin type A in the 6 months prior to study enrolment. The Melbourne Health Human Research Ethics Committee (HREC 2015.069) approved the study, and all participants provided voluntary written consent.

### Design and procedure

#### Clinical assessment

Clinical assessments included a general medical history, cerebellar assessment using the Scale for the Assessment and Rating of Ataxia (SARA)^49^ and neurological examination to assess the level of disability using the Expanded Disability Status Scale (EDSS) and the Cerebellar and Brainstem Functional System Scores (CBFSS).^50^

#### Speech assessment

We chose speech measures based on the current literature regarding speech analytics and neuroimaging in persons with multiple sclerosis^4,8,9^ (see Table 1 for an overview of all speech metrics included). Audacity version 2.1.2 (Free Software Foundation, Boston, MA, USA) was used for auditory–graphic manual screening of all speech data. During screening, pre- and post-task silences were deleted from the audio files ready for analysis. Speech analysis by Noffs *et al*.^4,9^ was mirrored in this study, though with fewer speech metrics to minimize the impact of multiple comparisons.

**Table 1:** Speech metrics included in analysis

Item	Type	Measures	Dysfunction indicated by
DDK rate	Acoustic	Number of syllables spoken per second: /pa-ta-ka/repeated for a minimum of 10 s.	Lower score
Read rate	Acoustic	Number of words read aloud per minute: Read a phonetically balanced passage aloud and the time taken was calculated.	Lower score
Log_10_naturalness	Perceptual	How typical the participant’s speech sounds: Assessed by a speech pathologist and otolaryngologist during reading and free speech.	Higher score (HC expected 0 score)
Vowel f0 CoV	Acoustic	Vowel frequency/pitch instability, voice control Sustained vowel/a:/for 10 s.	Higher score
Prolonged intervals	Perceptual	Total pause time during speech tasks: Percentage of total silence during reading and free speech.	Higher score
SARA speech sub-score	Perceptual	Ataxic dysarthria/cerebellar speech dysfunction: Speech is professionally assessed during normal conversation.	Higher score (HC expected 0 score)
Composite EDSS	Acoustic	Speech-related neurological dysfunction: Acoustic composite of pause percentage during free speech, read rate and vowel f0 CoV selected through a regression model for the EDSS.^4^	Higher score (HC expected 0 score)
Composite SARA	Acoustic	Speech-related cerebellar dysfunction: Acoustic composite of DDK rate, vowel f0 CoV, vowel energy CoV and pause percentage during free speech selected through a regression model for SARA.^9^	Higher score (HC expected 0 score)

CoV, coefficient of variation; DDK, diadochokinetic; EDSS, Expanded Disability Status Scale; SARA, Scale for the Assessment and Rating of Ataxia.

Acoustic speech assessment included the number of syllables pronounced per second (diadochokinetic rate, DDK rate), reading rate and vowel frequency instability [vowel f0 CoV (fundamental frequency coefficient of variation)]. Composite speech scores from the EDSS^4^ and SARA^9^ obtained through signal processing and calculated through forward stepwise regression were also included as acoustic measures. Acoustic analyses were conducted using VoiceSauce,^51^ MSP (Kay PENTAX, Lincoln Park, NJ, USA), Praat^52^ and MATLAB version 2018b (MathWorks, Natick, MA, USA; for more information, see Noffs *et al*.^4^).

Perceptual speech assessment included prolonged intervals and the speech sub-score from the SARA. We additionally assessed speech naturalness, a global measure of dysarthria used in patient groups such as multiple sclerosis, Parkinson’s disease and stuttering.^4,53,54^ Perceptual speech features were scored using a 4-point ordinal scale where 0 = normal and 4 = severely unnatural/unintelligible. One speech and language pathologist and one otolaryngologist (G.N.) rated the speech recordings blinded to the diagnosis of multiple sclerosis and multiple sclerosis disease severity. Both raters scored all samples from each participant simultaneously and independently. Only consensus scores were used in the analysis. Naturalness scores ≥ 1 were used as an indication of clinical dysarthria (for a more detailed description of speech analysis, see Noffs *et al.*^4,9^).

### MRI acquisition

Participants underwent 3-T MRI (MAGNETOM TrioTim, Siemens, Medical Systems, Erlangen, Germany) to obtain:

1. Sagittally acquired 3D fluid-attenuated inversion recovery (FLAIR): repetition time/echo time/inversion time = 5000/350/1800 ms; flip angle = 120°; in-plane resolution = 0.5 × 0.5 mm^2^; slice thickness = 1 mm.
2. Axially acquired contiguous 2D T_1_-weighted axial FLASH: repetition time/echo time = 250/2.5 ms; flip angle = 70°; in-plane resolution = 0.42 × 0.42 mm^2^; slice thickness = 5 mm.
3. Axially acquired 2D spin-echo echo-planar diffusion-weighted imaging: repetition time/echo time = 8600/120 ms; flip angle = 90°; in-plane resolution = 0.42 × 0.42 mm^2^; voxel size = 2.36 × 2.36 × 2.4 mm^3^; 1 *b*-value = 0 s/mm^2^; 30 directionally encoded diffusion-weighted images with gradient *b*-value = 3000 s/mm^2^. These specifications were chosen to balance acquisition time with the best possible signal-to-noise ratio. A higher *b*-value can improve apparent FD specificity by suppressing extra-axonal signal.^55^

### Lesion analysis

We created a lesion probability map to compare with regions showing axonal damage and loss as indicated by dMRI. Lesions were automatically segmented using the lesion prediction algorithm from the SPM12 Lesion Segmentation Toolbox. Lesion masks for each multiple sclerosis participant were generated from these lesion prediction algorithm outputs. The lesion maps were warped to the group white matter fibre orientation distribution (FOD) template using Advanced Normalisation Tools (ANTs) and then binarized and merged to form a single group level lesion probability map.

### Diffusion imaging analysis

dMRI data were processed with MRtrix3 using the FBA pipeline.^46,56^ Data pre-processing involved denoising, motion and eddy-current distortion correction. We then calculated the FOD for each voxel for each participant using multi-shell multi-tissue constrained spherical deconvolution. This provides an estimated axis of orientation for each fibre bundle passing through a voxel. We created a group white matter FOD template by non-linearly registering the white matter FOD map of each subject to a continually updated average template image. For each individual fibre population within a voxel, also known as a fixel,^57^ three fibre-specific measures were calculated—FD, fibre cross-section and FDC. Whole-brain probabilistic fibre tractography was performed on the group white matter FOD template to calculate fixel-to-fixel connectivity. To improve the reconstruction and reduce biases in tract densities, the tractogram was filtered to 2 million tracts using the SIFT2 algorithm.^58^

#### Identifying tracts of interest

**Figure 1:**
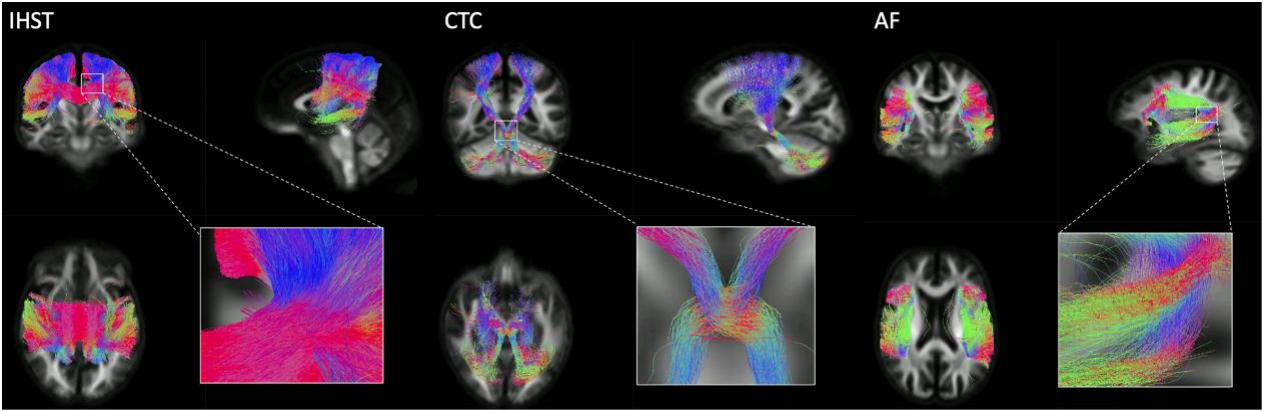
**White matter tracts included in FBA**. Tracts coloured according to direction: blue, superior–inferior; magenta, medial–lateral; green, anterior–posterior. AF, arcuate fasciculus; CTC, cerebello-thalamo-cortical tracts; IHST, interhemispheric sensorimotor tract.

Using previous research as guides, we identified specific white matter tracts to focus on the bilateral arcuate fasciculus and CTC pathways and the IHST (see Fig. 1). These tracts were selected using inclusion and exclusion regions of interest (ROIs), thresholded at 30% and combined to create one inclusion region per tract.

##### Interhemispheric sensorimotor tract

To select this tract, we included bilateral pre-central and post-central gyrus masks from the Harvard-Oxford Cortical Atlas (RRID:SCR_001476, provided by FSL v.6.0.3, FMRIB, Oxford, UK) that were registered to white matter FOD template space using ANTs (v2.3.1).^59^ Two coronal planes and one axial plane were used to exclude tracts running anterior or posterior. To exclude the thalamus, we used bilateral masks from the Harvard-Oxford Cortical Atlas registered to template space using ANTs.

##### CTC tract

For this tract, we used the 20 million tract tractogram, rather than using the SIFT2 filtered tractogram, to increase the number of tracts available for analysis. We manually created regions of interest for the bilateral superior cerebellar peduncle and red nucleus and used the Harvard-Oxford Atlas to mask the thalamus. Two sagittal planes were then used to prevent tracts running across hemispheres.

##### Arcuate fasciculus

For the arcuate fasciculus, we included bilateral BA44, BA45 and BA22, using masks of these regions from the Harvard-Oxford Cortical Atlas registered to white matter FOD template space using ANTs. To exclude tracts entering neighbouring regions and cerebellum, we used Harvard-Oxford Cortical Atlas masks of the brainstem, cerebellum, frontal pole, occipital cortex and corpus callosum. These were registered to white matter FOD template space using ANTs.

### Volumetric imaging analysis

FreeSurfer v6.0 (surfer.nmr.mgh.harvard.edu) was used to process MRI scans. Given the limited previous research on specific areas of the brain involved in dysarthria in multiple sclerosis, we opted for an exploratory analysis focusing on regions involved in motor speech production. We used the standard processing pipeline to segment and obtain the volume of each region of interest. The regions of interest selected for analysis include bilateral BA44, BA45 and BA22, corpus callosum, cerebellum and thalamus. Cerebellar segmentation into anterior, posterior and vermal regions was conducted using Automatic Cerebellum Anatomical Parcellation using U-Net Locally Constrained Optimization (ACAPULCO).^60^ Individual lesion maps were used for lesion filling of T_1_ structural images before volumetric analysis. Volumes were normalized as a percentage of total intracranial volume, which was calculated using FreeSurfer.

### Statistical analysis

Statistical analyses were run using SPSS 28 (IBM). Given the high skew on naturalness scores, with most participants scoring 0, we used a logarithmic transformation to increase normality. Despite this, the new log_10_naturalness scores still did not reach acceptable normality, and thus, non-parametric tests were used. Out of the remaining speech scores, only DDK rate scores were normally distributed. For DDK rate, we therefore used independent samples *t*-tests to assess group differences in brain volume and speech measures between multiple sclerosis and HC. Rather than transforming the remaining speech variables, we used independent samples Mann–Whitney *U*-tests to assess group differences. Then, Spearman correlations assessed the relationship between brain volume measures and speech metrics. Both group differences and correlations were corrected for false discovery rate to control for multiple comparisons. For tract-specific analysis, we used fixel-wise general linear models with connectivity-based fixel enhancement (family-wise error corrected *P* < 0.05) and non-parametric permutation testing. In a similar vein to our naturalness scores, we calculated the log of fibre cross-section for group analyses log_10_cross-section.^46^ Where significant group differences in fibre-specific measures were found, indicating white matter tract damage in persons with multiple sclerosis, we correlated the level of damage with speech metrics indicative of dysarthria using *P* < 0.05 adjusted for family-wise error rate.^57^ We controlled for age, sex and disease course (relapsing–remitting or progressive multiple sclerosis) for all correlations by including them as covariates in the general linear model.

## Results

### Participant demographics and clinical data

HCs had a mean age of 44.5 years, and 71% were female (see Table 2). Multiple sclerosis participants had a mean age of 47.8 years, 74.1% were female, and 59.2% had secondary progressive multiple sclerosis (one participant presented with primary progressive multiple sclerosis). The mean disease duration for persons with multiple sclerosis was 13.014 years. There were no significant differences in age or sex distribution between multiple sclerosis and control groups. Multiple sclerosis participants had an average EDSS score of 4.0 [interquartile range (IQR) = 2.5–6.0], a CBFSS of 2.0 (IQR = 2.0–3.0) and a SARA score of 11.048 (SD = 14.504). Multiple sclerosis participants performed significantly worse on all speech measures, with all differences remaining statistically significant following correction for multiple comparisons except vowel f0 CoV (see Table 2). A total of 19 multiple sclerosis participants presented with a naturalness score indicative of clinical dysarthria.

**Table 2:** Participant demographics, clinical measures of disease severity and cerebellar dysfunction and speech metrics

		HC	Multiple sclerosis	Test statistic	*P*	FDR-corrected *P*
Demographics	*N*	14	52	-	-	-
	Age (mean, SD)	44.476 (14.504)	47.847 (11.177)	0.248	0.403	-
	Sex (female %)	71.4	74.1	−1.163	0.124	-
Speech	SARA speech (mean, SD)	0.000 (0.000)	0.760 (0.970)	441.000^b^	<0.001	0.003
	Composite SARA (mean, SD)	−15.818 (3.547)	−9.454 (6.355)	203.000^b^	<0.001	0.003
	Composite EDSS (mean, SD)	−0.548 (0.566)	0.630 (1.276)	222.000^b^	<0.001	0.003
	DDK rate (mean, SD)	6.311 (1.086)	5.426 (0.992)	3.508^a^	<0.001	0.003
	Read rate (mean, SD)	3.868 (0.317)	3.430 (0.803)	509.000^b^	0.008	0.016
	Vowel f0 CoV (mean, SD)	0.835 (0.374)	1.148 (0.951)	540.000^b^	0.038	0.066
	Log10naturalness (mean, SD)	0.000 (0.000)	0.176 (0.211)	180.000^b^	0.001	0.003
	Prolonged intervals (mean, SD)	0.000 (0.000)	0.390 (0.662)	598.500^b^	0.004	0.009

CBFSS, Cerebellar and Brainstem Functional System Score; DDK, diadochokinetic; EDSS, Expanded Disability Status Scale; FDR, false discovery rate; f0 CoV, fundamental frequency coefficient of variation; SARA, Scale for the Assessment and Rating of Ataxia; SD, standard deviation.

^a^Independent samples *t*-test statistic.

^b^Mann–Whitney *U*-test statistic.

### dMRI group differences

**Figure 2:**
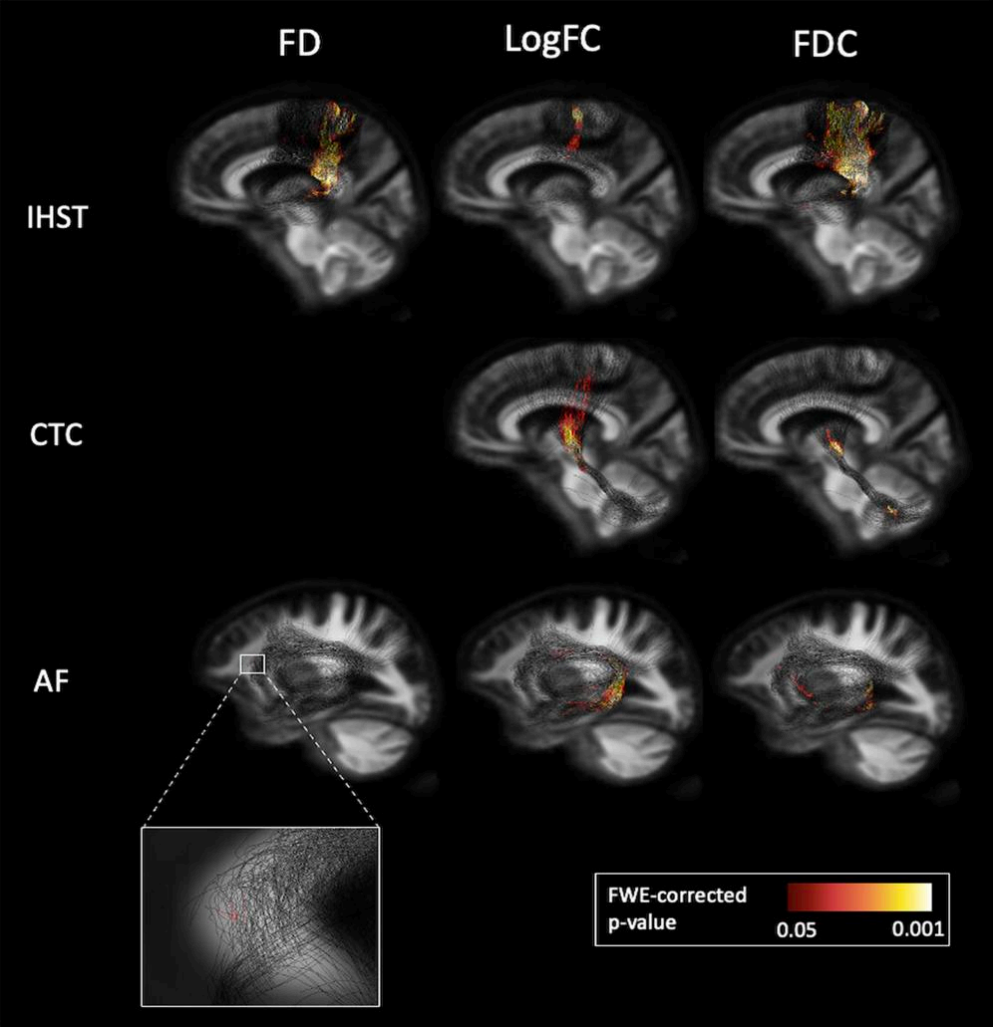
**Fibre-specific damage seen in persons with multiple sclerosis when compared with HC, determined by FBA**. AF, arcuate fasciculus; CTC, cerebello-thalamo-cortical tracts; FD, fibre density; FDC, fibre density × cross-section; IHSTs, interhemispheric sensorimotor tracts; LogFC, log_10_cross-section.

Tract-specific analysis of the IHST found reduced FD, log_10_cross-section and FDC in persons with multiple sclerosis compared with HC. This is mainly observed in the anterior mid-body and isthmus of the corpus callosum and connected corona radiata extending to the primary sensorimotor cortices. Further, we saw reduced log_10_cross-section and FDC in the CTC in persons with multiple sclerosis compared with controls, specifically within the left superior cerebellar peduncle and right thalamus. Finally, a reduced FD, log_10_cross-section and FDC in the arcuate fasciculus, in regions connecting to and surrounding BA44/45 and BA22, was found in persons with multiple sclerosis compared with HC (Fig. 2).

### Intersection of white matter lesions and fibre-specific damage

**Figure 3:**
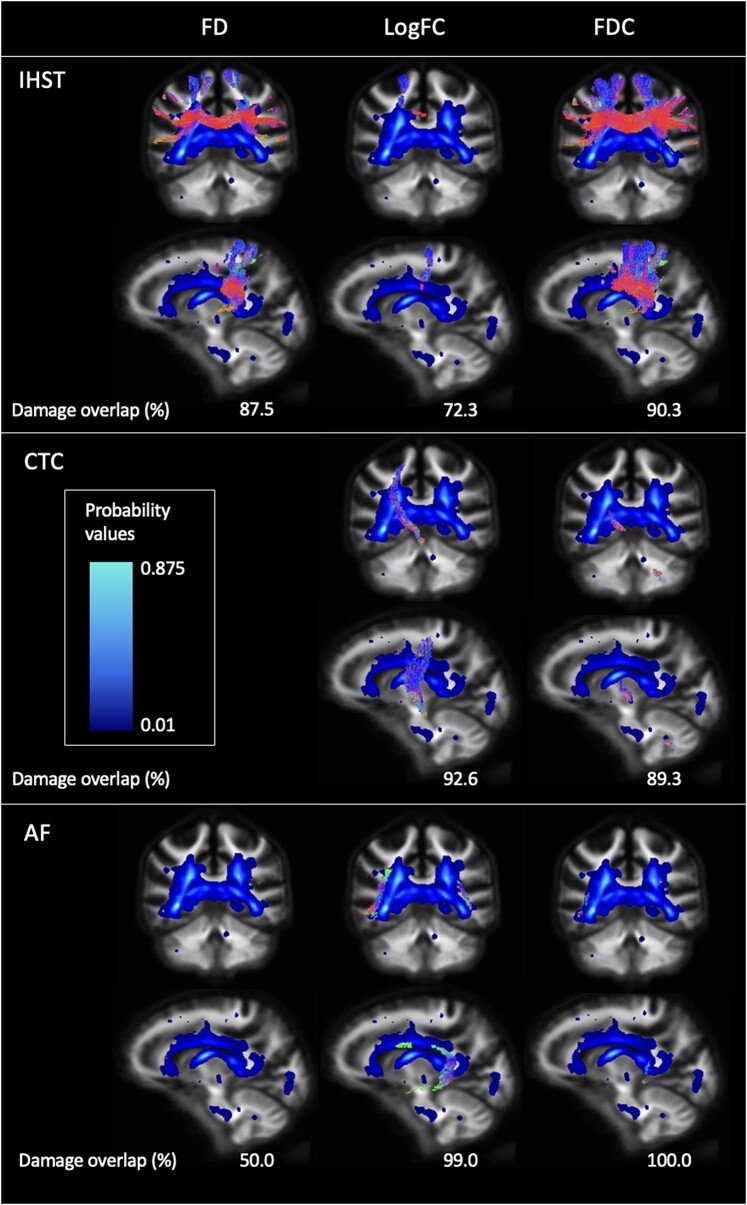
**Lesion probability map overlap with damage in tracts of interest**. Tracts coloured according to direction: blue, superior–inferior; red, medial–lateral; green, anterior–posterior. Low lesion location probability is seen in navy, and higher location probability is shown in light blue. AF, arcuate fasciculus; CTC, cerebello-thalamo-cortical tracts; FD, fibre density; FDC, fibre density × cross-section; LogFC, log_10_cross-section; IHSTs, interhemispheric sensorimotor tracts.

We examined the overlap between the lesion probability map and areas of axonal damage within the tracts of interest in persons with multiple sclerosis. The maximum probability was 87.5%, indicating the presence of lesions in the same location in 87.5% of multiple sclerosis participants. The probability map intersected with 56.5% of the IHST, 59.3% of the CTC and 63.2% of the arcuate fasciculus (Fig. 3).

### Correlations between fibre-specific measures of axonal loss, clinical and speech metrics

**Figure 4:**
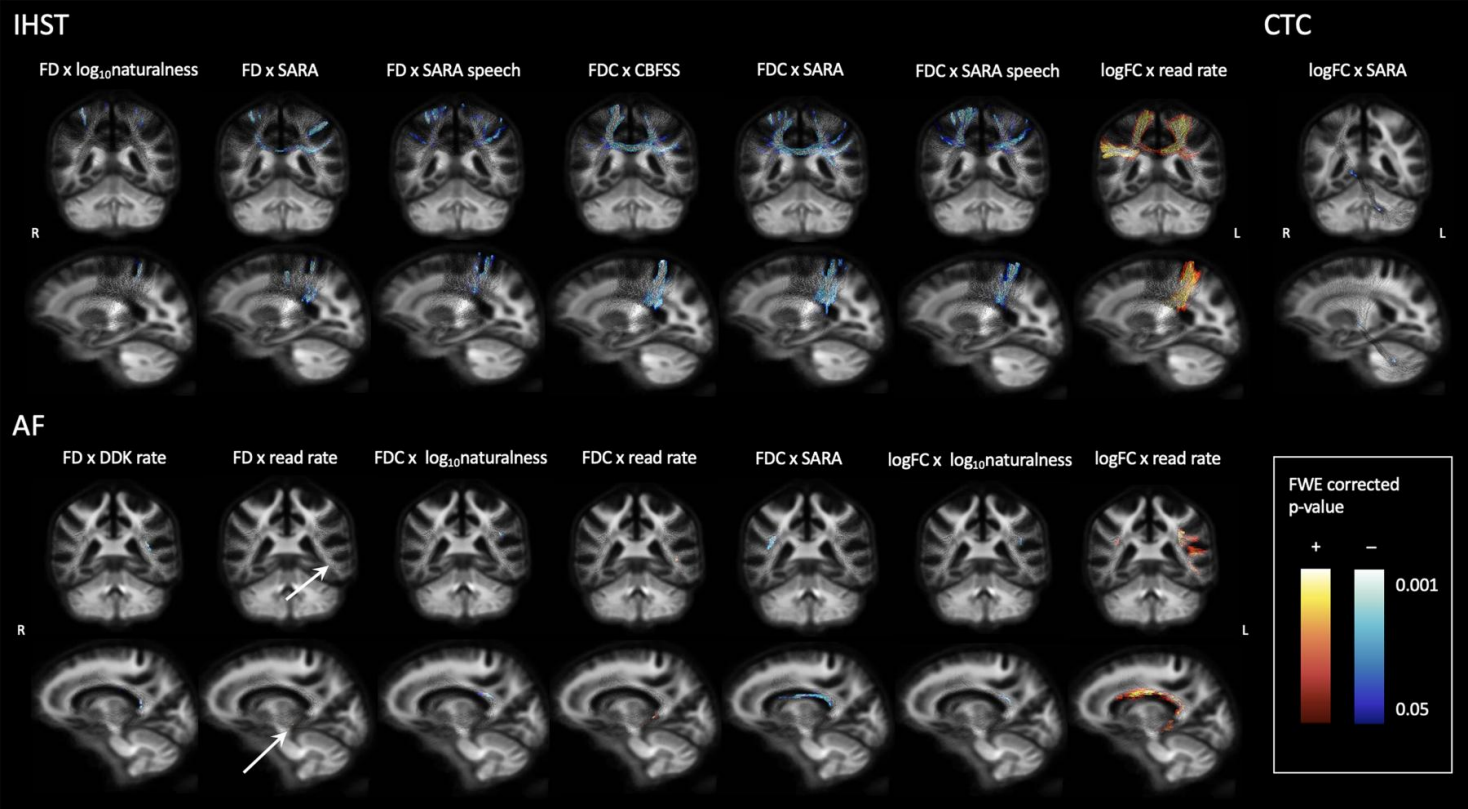
**Fibre-specific measures of axonal damage correlate with cerebellar and speech metrics in persons with multiple sclerosis**. Colour indicates direction of correlation, where warm colours show positive correlation and cool colours show a negative correlation. Correlations were controlled for age, sex and multiple sclerosis disease course (progressive or relapsing–remitting). *P* < 0.05 adjusted for family-wise error rate. AF, arcuate fasciculus; CBFSS, Cerebellar and Brainstem Functional System Score; CTC, cerebello-thalamo-cortical tracts; DDK, diadochokinetic; FD, fibre density; FDC, fibre density × cross-section; f0 CoV, fundamental frequency coefficient of variation; IHST, interhemispheric sensorimotor tract; LogFC, log_10_cross-section; SARA, Scale for the Assessment and Rating of Ataxia.

Log_10_naturalness and read rate correlated with FD, log_10_cross-section and FDC in the IHST and arcuate fasciculus. CBFSS, SARA and SARA speech scores correlated with axonal loss in the IHST and CTC tracts (Fig. 4). While we observe quite localized associations between FBA metrics and speech measures, particularly in the CTC tracts and arcuate fasciculus, uncorrected *P*-value maps (see Supplementary Fig. 1) showed a broader effect.

### Volumetric group differences

Independent samples group comparisons showed persons with multiple sclerosis had a lower volume in the corpus callosum (*t* = 3.293, *P* = 0.016) and bilateral thalamus (left *t* = 2.386, *P* = 0.019; right *U* = 169.000, *P* = 0.009). We also found a higher volume of the left posterior cerebellum (*t* = 3.625, *P* < 0.003) in persons with multiple sclerosis compared with HC. There were no significant group differences in brain volume in the other selected regions. All group comparisons in brain volumetrics can be seen in Supplementary Table 1.

### Correlations between volumetrics and speech metrics

When controlling for age, sex and disease course, composite SARA scores correlated with bilateral BA45 volume (left = −0.721, *P* = 0.028; right = −0.813, *P* = 0.008). However, following correction for false discovery rate, neither of these correlations remained significant. We also saw trends in the relationships between left anterior cerebellum volume and both EDSS (0.624, *P* = 0.072) and SARA (0.644, *P* = 0.061) scores. Bilateral BA45 volume also showed a trend with DDK rate (left = 0.638, *P* = 0.065; right = 0.645, *P* = 0.061). Finally, right BA45 volume and composite EDSS scores showed a trending relationship (0.658, *P* = 0.054; see Supplementary Table 2 for all correlations).

## Discussion

The present study used structural and diffusion neuroimaging techniques to assess the neural substrates of speech dysfunction in persons with multiple sclerosis. We found substantial indications of axonal damage in persons with multiple sclerosis in three major motor and speech tracts—the IHST, CTC and arcuate fasciculus. It was further discovered that IHST and arcuate fasciculus damage correlates with measures of dysarthria in persons with multiple sclerosis and that ataxia severity indicative of cerebellar dysfunction in multiple sclerosis correlates with diffusion markers of axonal injury and loss in the IHST, CTC and arcuate fasciculus. These findings juxtapose those of volume reductions in motor speech regions of the brain, which were minor and did not correlate significantly with any measures of disease severity, cerebellar or speech dysfunction.

### Marked axonal damage in motor speech–associated white matter tracts in multiple sclerosis

Analysis of diffusion data exposed both global and tract-specific indicators of axonal damage and loss in persons with multiple sclerosis when compared with HC, as signified by reduced FD, log_10_cross-section and FDC. Our multiple sclerosis cohort showed white matter tract atrophy primarily around the lateral ventricles. This builds on past research that found increased mean diffusivity in the same region, indicative of degeneration.^61^ Previous studies also show a significant decrease in FD and FDC between persons with multiple sclerosis and HCs^62^ and in persons with multiple sclerosis over time.^28^ Further, the diffuse reduction of log_10_cross-section, suggestive of widespread white matter atrophy, has been observed in persons with multiple sclerosis, with a greater reduction associated with greater disease progression.^32^ Our findings thus support the current literature regarding global white matter tract damage in multiple sclerosis. When exploring fibre-specific damage, we found lower log_10_cross-section and FDC in the IHST, CTC and arcuate fasciculus in persons with multiple sclerosis compared with HC. Lower FD was seen in the IHST and arcuate fasciculus, but not in the CTC. Our findings of axonal damage in the IHST and CTC mirror previous work in persons with multiple sclerosis.^42^

### Correlations between white matter tract damage and clinical and speech metrics

We discovered bilateral IHST damage to negatively correlate with cerebellar dysfunction (CBFSS and SARA). Additionally, cerebellar dysfunction, measured by the SARA score, was associated with axonal loss in the CTC, specifically in the right thalamus, the left superior cerebellar peduncle and the left arcuate fasciculus. We found higher perceptual speech dysfunction (naturalness) scores to correlate with axonal damage in the right IHST and left arcuate fasciculus approaching BA22, or Wernicke’s area. While there are no comparative results in a multiple sclerosis cohort, research into traumatic brain injury has similar findings. Liégeois *et al.*^63^ found that speech impairment in dysarthric patients is associated with reduced fractional anisotropy in the left arcuate fasciculus and with reduced arcuate fasciculus and corpus callosal volume. However, past research has also found changes in diffusion measures but not corpus callosal volume relating to language lateralization and word generation.^64^ Likewise, here, we have shown that while there was no significant relationship between macrostructural corpus callosal volume reduction and motor speech dysfunction, microstructural white matter tract damage within the corpus callosum correlates with dysarthria measures in persons with multiple sclerosis. Moreover, a study investigating the motor region of the corpus callosum in early multiple sclerosis found microstructural abnormalities, as evidenced by decreased fractional anisotropy, before macrostructural lesions and atrophy were detectable.^65^ Longitudinal research may therefore shed light onto whether this relationship between motor speech production and corpus callosal damage progresses over time.

Moreover, we discovered that reading rate was associated with axonal loss throughout the left arcuate fasciculus. A slower reading rate has previously been highlighted in persons with multiple sclerosis with dysarthria.^66^ Both speech naturalness and reading rate have been associated with higher levels of disability (EDSS and CBFSS) and cerebellar dysfunction (CBFSS and SARA) in persons with multiple sclerosis.^9^ Further, a 2019 study used a traditional dMRI measure, fractional anisotropy, to establish the connection between damage in the left arcuate fasciculus and reduced verbal fluency in persons with multiple sclerosis.^33^ Damage of the left arcuate fasciculus correlating with lower reading rate thus provides insight into the pathophysiology of dysarthria in persons with multiple sclerosis. Contrasting this, the correlation between higher DDK rate and lower FD in the left arcuate fasciculus near BA22 is opposite to what was expected. It is possible that this is a type II error due to sample size, and further studies in larger cohorts are needed.

### Overlap of white matter tract damage with lesion location

Strik *et al.*^42^ found minor overlaps of lesion location and motor tract damage across the IHST, CTC and cortico-spinal tract, although this study looked at minimally disabled persons with multiple sclerosis. Conversely, we focused on persons with multiple sclerosis with established disease and moderate disability and found probable lesion location to substantially overlap with areas of damage within motor speech white matter tracts. Out of our three tracts of interest, damaged areas of the arcuate fasciculus had the highest percentage overlap with the lesion probability map. Impairment of speech production has been associated with lesion load in the left arcuate fasciculus.^35^ This relationship has been demonstrated in the left superior arcuate fasciculus and inferior cerebellar peduncle, showing alterations in diffusion patterns not only at the site of the lesion but also along the tracts.^67^

A recent study found a relationship between diffusion measures (radial diffusivity) and the presence of slowly expanding lesions in persons with multiple sclerosis.^68^ Slowly expanding lesions have been linked to multiple sclerosis progression in both relapsing–remitting^69,70^ and progressive^69,71^ forms of the disease. Lesion expansion also contributes more to brain atrophy and increase in EDSS scores compared with the impact of new lesions.^70^ While we were unable to measure this in our cohort, it is important to keep in mind for future studies that slowly expanding lesions may be a factor in the overlap between lesion location and white matter tract damage.

### Minor volumetric changes in motor speech areas in multiple sclerosis

Previous research has revealed that whole cerebrum grey and white matter reduction or whole cerebellum grey and white matter reduction correlate with speech measures of dysarthria in persons with multiple sclerosis.^4,8^ We expanded on these findings by focusing on specific motor speech cortical and subcortical regions of the cerebrum and by sectioning the cerebellum into anterior, posterior and vermal regions. Thalamic atrophy is well described in multiple sclerosis and is linked to increased upper limb tremor and compromised ambulation in persons with multiple sclerosis,^24,72^ indicating a relationship between thalamic damage and reduced motor performance. Additionally, a 2007 study found that deep brain stimulation to the thalamus impacts production of speech sounds and articulation in persons with multiple sclerosis.^73^ We found lower thalamic volume in persons with multiple sclerosis compared with HC, replicating previous findings. However, there was no correlation between thalamic atrophy in persons with multiple sclerosis and worse motor speech tasks or cerebellar functioning.

Similarly, we measured lower corpus callosal volume in persons with multiple sclerosis, indicative of atrophy, consistent with the reported literature.^21,22,26^ Corpus callosum damage and atrophy relate to verbal fluency in persons with multiple sclerosis.^26^ However, we could not confirm these findings in our study, with no correlation between corpus callosal volume and any of our speech metrics. This could be due to the nature of the tasks we used compared with other studies. While there are motor aspects to verbal fluency,^74^ the study by Pozzilli *et al.*^6^ used more cognitive tasks to assess verbal fluency. The speech metrics included in our study focus only on the motor aspects of speech.

We did not find reductions in volume in other brain regions associated with speech production such as the right cerebellum and left BA44/BA45^9-11,35,75-79^ although left anterior cerebellar volume showed non-statistically significant trends with both EDSS and SARA scores in persons with multiple sclerosis. The lack of significant correlations here may be due to the sample size or level of disease progression in our cohort, which could mean that the measures needed to be higher powered or more sensitive to minor changes in brain volume to establish any significant differences, particularly in small regions such as BA44/BA45. Further, cortical areas such as BA44/BA45 are not as commonly impacted by multiple sclerosis than other regions.^80^ We did however find an association between bilateral BA45 volume and the acoustic composite measure of cerebellar speech dysfunction and SARA score measured dysarthria in persons with multiple sclerosis.^9^ While this correlation did not survive correction for multiple comparisons, it indicates a trend worth exploring. BA45 volume also showed a relationship with syllable repetition rate (DDK rate) and speech-related neurological dysfunction (composite EDSS scores), although these only approached significance. A 2007 study found reciprocal connections between BA44/BA45 and the cerebellum, suggesting that these regions are connected in articulatory processing.^81^ Further investigation into this relationship in the context of dysarthria in multiple sclerosis is consequently advised.

## Limitations and considerations

Multiple sclerosis participants had moderately high EDSS scores and moderately long disease duration, so the findings may not be generalizable to all persons with multiple sclerosis. While we had an adequate sample size for this study with a total of 52 persons with multiple sclerosis involved, the number of participants did not allow for exploring subgroups of multiple sclerosis patients, such as different disease severity or level of cerebellar dysfunction. It would be interesting for future research to study persons with multiple sclerosis at different disease stages or with different levels of cerebellar and speech functioning. Additionally, the percentage of tract damage implied by diffusion analysis was small, and it is possible that family-wise error rate correction for multiple comparisons led to an underestimation of the extent of axonal damage. While diffusion neuroimaging provides us with a tool for assessing white matter tract damage, it does not directly image the tissue. Rather, dMRI gives indirect measurements of the diffusion of water molecules throughout these tracts.^82^ Research has been conducted regarding post-mortem validation of diffusion neuroimaging.^82^ However, these have often been limited by sample size or having separate living dMRI and post-mortem subjects. To our knowledge, no research has been conducted validating fixel-based diffusion metrics against post-mortem measures of axonal damage and loss. Thus, we cannot confidently say what each diffusion metric used in this study measures in terms of structural integrity, demyelination and axonal injury. We also did not evaluate the presence of slowly expanding lesions. Given the role of slowly expanding lesions in both brain atrophy and changes in diffusivity, their presence within this cohort could explain the degree of overlap between lesion location and white matter injury. We also saw a higher volume of the left posterior cerebellum in persons with multiple sclerosis than in HC. This diverges from previous findings, where lower cerebellar volume is linked to multiple sclerosis disease progression,^23,25,83^ and is most likely a measurement error. Further, our multiple sclerosis cohort showed minimal signs of clinically significant dysarthria, as measured through naturalness scores. While acoustic speech metrics can detect subclinical dysarthria, the findings of this study may be more applicable to patients with mild-to-moderate clinical dysarthria. Future research in a cohort with more varying levels of speech dysfunction and clinical dysarthria would demonstrate the relationships between volumetric and diffusion abnormalities and dysarthria to a greater extent. On this note, the speech analysis included speech domains of timing and voice control but did not include a measure of voice quality. Noffs *et al*.^4^ included cepstral peak prominence—which measures voice strain—in their analysis and found it to be correlated with disease severity as measured by the EDSS. A measure of voice quality, such as cepstral peak prominence, would be a good addition to analysis of diffusion and speech metrics in multiple sclerosis. There are also other speech deficits known to be common in multiple sclerosis that have not been represented in acoustic metrics thus far, including a low accuracy of consonants. Inclusion of an appropriate measure of consonant accuracy may strengthen future findings.

Finally, this study has focused on the motor aspects of speech production. It is crucial to make note of the significant cognitive contribution to speech dysfunction in multiple sclerosis.^33,84,85^ While we have attempted to include speech measures indicative of motor speech dysfunction and focused on white matter tracts specifically involved in the motor aspects of speech, it is challenging to remove cognition from the equation completely. Thus, we cannot say with certainty that our results reflect purely motor dysfunction. We suggest future studies either control for the cognitive aspect of speech production or broaden the scope of the research to include cognition and the impact of cognitive dysfunction on speech and dysarthria in multiple sclerosis.

## Conclusion

This study highlights the impact of white matter tract injury on motor speech dysfunction in persons with multiple sclerosis. Specifically, we found substantial associations between IHST and arcuate fasciculus damage and dysarthria in persons with multiple sclerosis without any significant relationship between speech metrics and volume of motor speech regions of the brain connected by these tracts. Further, we found greater axonal damage within these tracts than volumetric change in motor speech regions of the brain. This suggests that the pathogenic injury that underpins dysarthria in persons with multiple sclerosis is a microstructural, tract-level damage rather than macrostructural atrophy of motor speech areas. However, further research is required to confirm this. We suggest future studies focus on longitudinal research to determine whether speech metrics such as naturalness, SARA speech sub-score and reading rate could be used to monitor disease progression in persons with multiple sclerosis. Further, we recommend longitudinal analyses into how the relationship between fibre-specific and volumetric measures of atrophy develops as multiple sclerosis progresses and how this relates to speech dysfunction and clinical disability. This would give researchers and clinicians a greater understanding of the underlying mechanisms of dysarthria in persons with multiple sclerosis. Analysis comparing multiple sclerosis subgroups and persons with multiple sclerosis with differing levels of speech dysfunction would also be beneficial.

## Supplementary Material

fcae177_Supplementary_Data

## Data Availability

The data that support the findings of this study are available from A.v.d.W upon reasonable request.
