# Supplementary material for: Volumetric and diffusion MRI abnormalities associated with dysarthria in multiple sclerosis
Source: Brain Commun. 2024 May 21;6(3):fcae177. doi: 10.1093/braincomms/fcae177 (PMC11154149; doi:10.1093/braincomms/fcae177)
Supplement: fcae177_Supplementary_Data [file fcae177_supplementary_data.docx]

**Supplementary Table 1: Group differences in brain volume by region.**

L: left hemisphere; R: right hemisphere; BA: Brodmann Area; ^Independent samples t statistic; °Mann-Whitney U statistic.

| Brain region | Average (HC) | Average (MS) | Test statistic | *p* | FDR-corrected *p* |
| --- | --- | --- | --- | --- | --- |
| BA44 L (Broca’s area) | 0.295 | 0.290 | °310.000 | .585 | .608 |
| BA44 R | 0.254 | 0.246 | °292.000 | .399 | .486 |
| BA45 L (Broca’s area) | 0.239 | 0.237 | ^0.154 | .439 | .496 |
| BA45 R | 0.272 | 0.272 | °340.000 | .960 | .960 |
| BA22 L (Wernicke’s area) | 0.794 | 0.752 | °256.000 | .150 | .244 |
| BA22 R | 0.741 | 0.719 | ^0.990 | .164 | .251 |
| Total cerebellum | 0.088 | 0.087 | ^0.343 | .366 | .476 |
| Cerebellum anterior L | 0.005 | 0.005 | ^-0.844 | .201 | .290 |
| Cerebellum anterior R | 0.005 | 0.005 | °299.000 | .467 | .506 |
| Cerebellum posterior L | 0.028 | 0.032 | ^-3.625 | <.001 | .003 |
| Cerebellum posterior R | 0.028 | 0.027 | ^0.226 | .411 | .486 |
| Cerebellum vermis | 0.004 | 0.004 | °284.000 | .329 | .450 |
| Thalamus L | 0.487 | 0.430 | ^2.386 | .010 | .019 |
| Thalamus R | 0.465 | 0.405 | °169.000 | .004 | .009 |
| Corpus callosum | 0.262 | 0.218 | °182.000 | .008 | .016 |

**Supplementary Table 2: Spearman correlations between speech metrics and brain volumetrics.**

|  | SARA | CBFSS | EDSS | SARA  speech | Composite  SARA | Composite  EDSS | DDK rate | Read  rate | Vowel  F0 CoV | Log10  Naturalness | Prolonged  intervals |
| --- | --- | --- | --- | --- | --- | --- | --- | --- | --- | --- | --- |
| BA44 L | 0.249 | -0.033 | 0.252 | -0.132 | -0.427 | -0.230 | 0.516 | -0.013 | 0.065 | -0.305 | -0.381 |
| BA44 R | 0.467 | -0.173 | 0.455 | 0.048 | -0.058 | 0.077 | 0.301 | -0.019 | -0.439 | -0.067 | -0.027 |
| BA45 L | 0.171 | 0.036 | 0.060 | -0.137 | **-0.721** | -0.520 | 0.638 | 0.490 | -0.152 | -0.409 | -0.244 |
| BA45 R | 0.129 | -0.141 | -0.041 | -0.295 | **-0.813** | -0.658 | 0.645 | 0.580 | -0.415 | -0.238 | -0.441 |
| BA22 L | 0.057 | 0.058 | 0.108 | 0.097 | -0.488 | -0.281 | 0.572 | 0.103 | -0.197 | -0.392 | -0.111 |
| BA22 R | 0.066 | 0.003 | 0.044 | 0.144 | -0.583 | -0.430 | 0.587 | 0.206 | -0.288 | -0.269 | -0.224 |
| Total Cerebellum | 0.428 | -0.083 | 0.454 | 0.185 | 0.308 | 0.264 | -0.245 | -0.306 | 0.477 | -0.136 | -0.199 |
| Cerebellum anterior L | 0.644 | 0.358 | 0.624 | 0.589 | 0.275 | 0.092 | -0.513 | -0.128 | 0.415 | 0.144 | -0.146 |
| Cerebellum posterior L | 0.492 | 0.322 | 0.525 | 0.483 | -0.015 | -0.183 | -0.353 | -0.167 | 0.079 | 0.246 | -0.494 |
| Cerebellum anterior R | 0.296 | 0.246 | 0.421 | 0.273 | 0.293 | 0.295 | -0.275 | -0.299 | 0.647 | -0.308 | 0.034 |
| Cerebellum posterior R | 0.364 | 0.083 | 0.458 | 0.292 | -0.012 | 0.009 | -0.004 | -0.291 | 0.226 | -0.170 | -0.335 |
| Cerebellar vermis | -0.004 | 0.149 | -0.064 | 0.008 | -0.355 | -0.383 | -0.023 | 0.371 | 0.381 | -0.390 | -0.273 |
| Thalamus L | 0.386 | 0.131 | 0.306 | 0.099 | -0.530 | -0.384 | 0.505 | 0.266 | -0.295 | -0.107 | -0.225 |
| Thalamus R | 0.309 | -0.002 | 0.145 | 0.232 | -0.593 | -0.522 | 0.508 | 0.422 | -0.470 | -0.016 | -0.233 |
| Corpus callosum | -0.024 | 0.072 | 0.110 | 0.255 | -0.201 | -0.001 | 0.471 | -0.032 | -0.610 | -0.202 | 0.310 |

L: left hemisphere; R: right hemisphere; BA: Brodmann Area; **p*<.05. SARA: Scale for the Assessment and Rating of Ataxia; DDK: diadochokinetic; f0 CoV: fundamental frequency coefficient of variation. Values in bold have an uncorrected *p*<.05.

**Supplementary Fig. 1: Speech and diffusion metric correlations in pwMS (uncorrected)**

Colour indicates direction of correlation - blue: negative, orange: positive. CBFSS: Cerebellar and Brainstem Functional System Score; SARA: Scale for the Assessment and Rating of Ataxia; DDK: diadochokinetic; f0 CoV: fundamental frequency coefficient of variation; log10Nat: log_10_Naturalness;

FD: fibre density; FC: fibre cross-section; FDC: FD x FC.


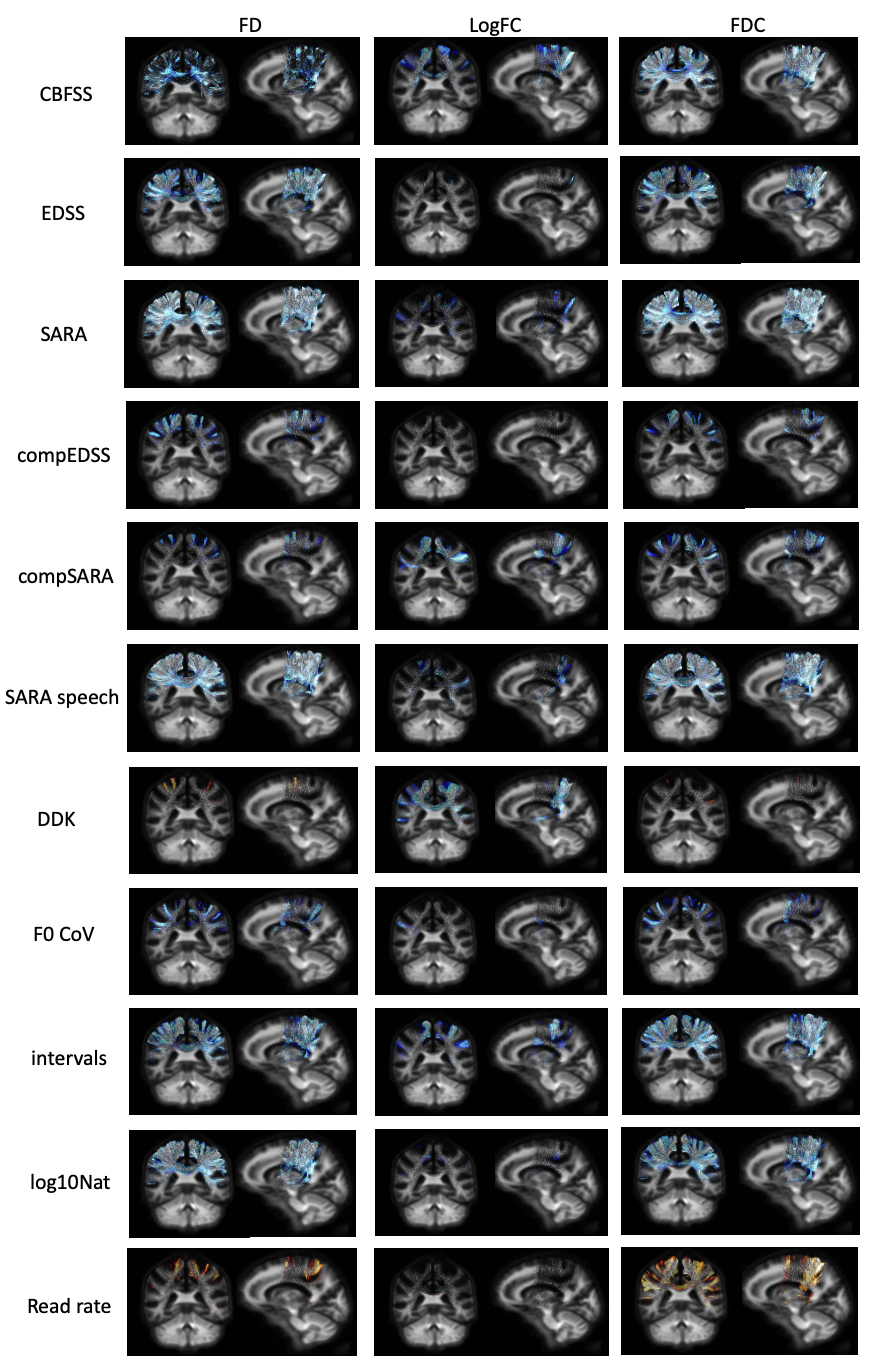
1A. INTERHEMISPHERIC SENSORIMOTOR TRACTS


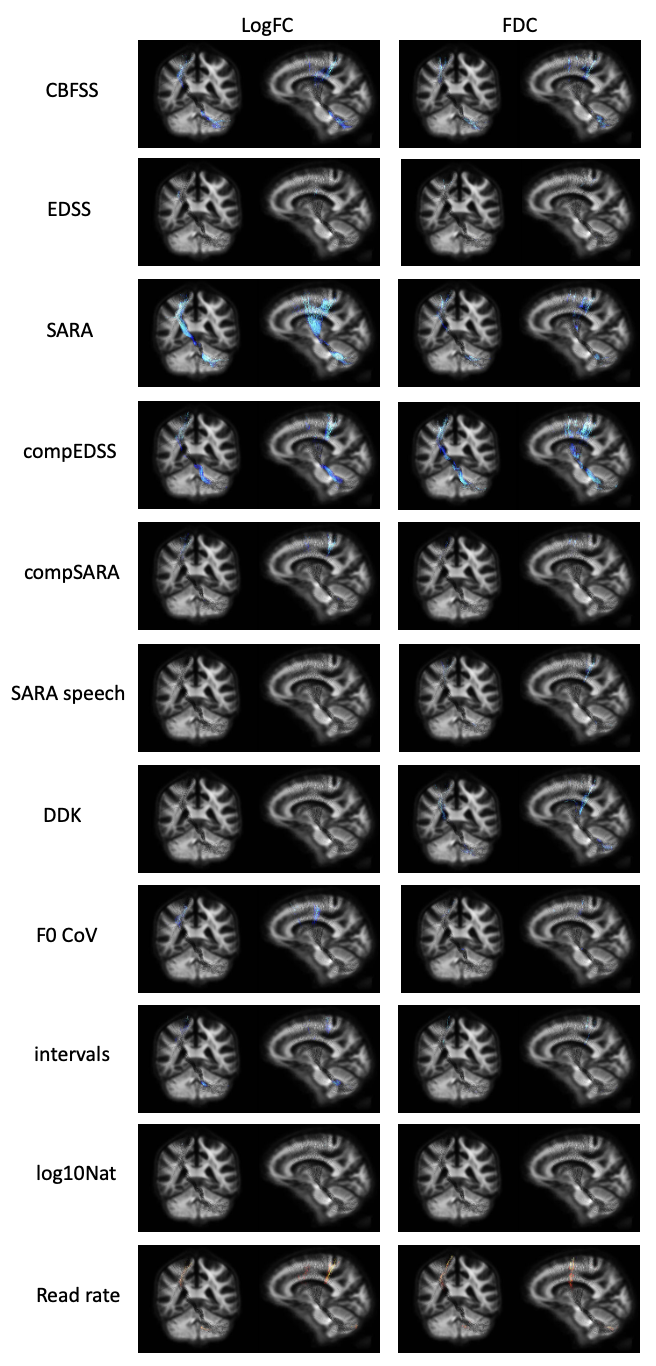
1B. CEREBELLO-THALAMO-CORTICAL TRACTS


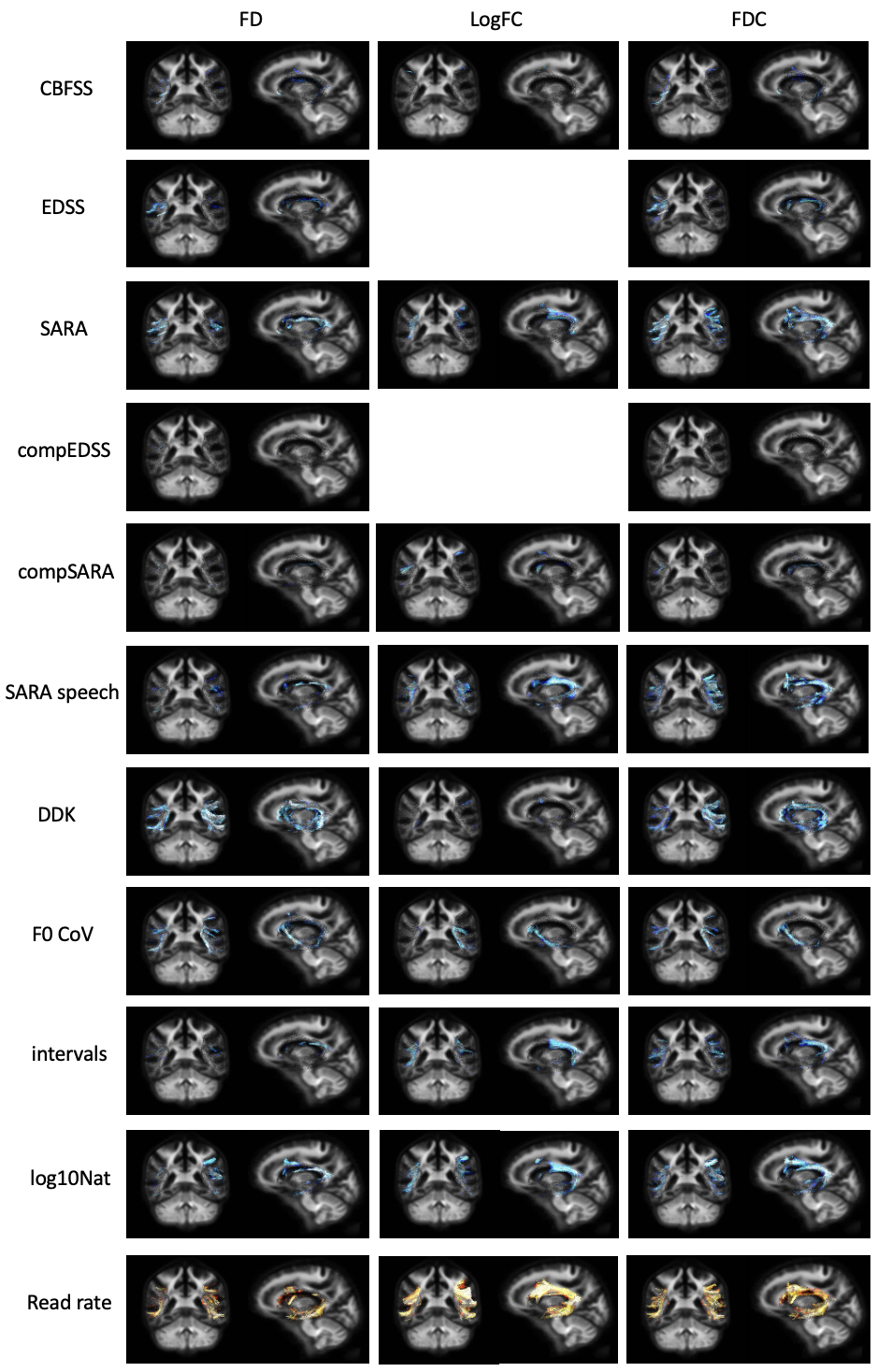
1C. ARCUATE FASCICULUS
